# Magnitude of work-related musculoskeletal disorders and its associated factors among Ethiopian nurses: a facility based cross-sectional study

**DOI:** 10.1186/s12891-024-07479-x

**Published:** 2024-06-07

**Authors:** Amsalu Nemera, Mergitu Eliyas, Teferra Likassa, Milion Teshome, Bulti Tadesse, Yonas Gurmu Dugasa, Meseret Robi Tura

**Affiliations:** 1 Department of Nursing, College of Health Sciences and Referral Hospital, Ambo University, Ambo, Ethiopia; 2 Department of Nursing, College of Medicine & Health Sciences, Ambo University, P. O. Box 19, Ambo, Ethiopia

**Keywords:** *Magnitude*, *Musculoskeletal disorders*, *Nurses*, *Work*

## Abstract

**Introduction:**

Work-related musculoskeletal disorders have a greater negative impact on nurses’ well-being, both physically and emotionally. Because of this issue, nurses take excessive leave, are absent from work, quit their profession, and have a lower quality of life. This leads to an increase in human and economic losses to health care institutions. However, there is a paucity of information about work-related musculoskeletal disorders among nurses in the study area.

**Objective:**

This study aimed to assess the magnitude of work-related musculoskeletal disorders and its factors associated among nurses in Hospitals of West Shoa Zone, Central Ethiopia, 2021.

**Materials and methods:**

A cross-sectional facility-based study was conducted with 406 systematically selected nurses from March 2021 to June 2021. A simple random sampling was used to select study subjects. Data were gathered through face-to-face interviews. The data has been cleaned and checked, entered Epi Data 3.1, and exported into Statistical Package for Social Sciences software version 25 for further analysis. Bivariable and multiple logistic regressions were used to determine the factors associated with outcome variable. The level of statistical significance was reported to be less than 0.05.

**Results:**

The prevalence of work-related musculoskeletal disorders was 73.8% and the highest report 247 (62.2%) was seen in the lower back which was followed by the neck 182 (45.8%) and elbow 126 (31.7%), while the lowest affected body part was ankle 46(11.6%). Working in the medical ward (COR = 5.91, 95%CI: 2.53–13.79), surgical ward (COR = 9.63, 95%CI: 3.64–15.51), and operation room theater (COR = 6.02, 95%CI: 2.37–15.27) had higher risk to work-related musculoskeletal discomfort as compared to those who were worked in outpatient department. Nurses those experienced bending or twisting back as a problem during work 2.33 times more likely to have higher risk WMSDs as compared to those who didn’t perceive it with (COR 2.33, 95% CI: 1.47–3.71).

**Conclusion:**

In this study, the prevalence of work-related musculoskeletal disorders among nurses was high. Working in room/ward, working in malposition, have no on job training to prevent work-related musculoskeletal disorders and bending or twisting back during work were identified as associated factors. Since work-related musculoskeletal disorders are preventable, educating the staff about the use of proper body mechanics and modifying the work environment is essential.

## Introduction

Work-related musculoskeletal disorders (WMSDs) are defined as discomforts that the worker encounters on their neurovascular and musculoskeletal systems, which are exacerbated by the setting in which they work [1, 2]. One of the major problems facing developed countries is work-related musculoskeletal diseases (WMSD), which are caused by repeated and stressful work environments. The symptoms of WMSDs includes stiff joints, dull pains, swelling, and recurrent pain. Overuse injuries, cumulative trauma disorders, and repetitive strain injuries (RSIs) are among terms used to describe musculoskeletal problems associated with the workplace [3–5]. WMSD problems occur frequently especially in the profession of nursing [6]. Research indicates that WMSDs are highly prevalent in nursing staff, with prevalence in the range of 60 to 95% [7, 8]. Physical and psychosocial variables are linked to musculoskeletal illnesses related to the workplace. Awkward postures, prolonged standing or sitting, repetitive manual labor, and manual handling of objects, including patient handling, are examples of physical factors. Organizational and job content variables are considered psychological factors [9]. High workloads, strict deadlines, and a lack of control over work and working techniques are all factors that are related to the content of a job. Organizational features address risk factors such as financial demoralization, bad work/rest cycle, poor community support, and inadequate communication with supervisors and coworkers [10, 11]. Other associated factors may include individual–related factors comprising demography and lifestyle factors [9, 12–13]. Age, body mass index, and gender are among the demographic variables. Lifestyle factors that may contribute to the development of MSDs include those relating to drinking, smoking, exercise, sports, leisure activities, housework, and other responsibilities that individuals play outside of the job [14].

Several studies have been carried out globally to ascertain the impact of WMSDs on different healthcare workers [2, 3, 15]. Research from Australia and Canada has revealed that WMSDs have such a significant negative social and economic impact that preventative measures must be developed [16]. Study from a variety of African countries, namely Egypt and Nigeria, indicated that WMSDs affect workers’ quality of life and productivity [14, 17]. WMSDs are the most expensive form of work disability, attributing about 40% of all costs toward the treatment of work-related injuries [17]. WMSDs continue to be a major occupational health problem to both government agencies and the private industry work environment [17]. It presents a significant socioeconomic and public health issue for healthcare professionals globally [18]. WMSDs are reported to be the most common cause of disability and occupational health problems among nurses in most hospitals in many countries worldwide [13]. The nursing profession is regarded as the profession with a high prevalence of MSDs compared to other occupations [19]. The nursing profession is a very demanding job, both physically and emotionally which makes nurses more susceptible to WMSDs [18].

Work-related musculoskeletal disorders are a significant public health issue that have an influence on nursing performance at work and have a range of social, cultural, and financial consequences. Low back pain (LBP) is the most common musculoskeletal disorder related to the incapacity of nurses, and it is estimated that 200,000 nurses suffer each year costing the National Health Service approximately £45 million [20, 21]. WMSD problems are common and most prevalent in the nursing profession [6]. Studies have shown that the prevalence rate of WMSDs in nurses is high ranging between 60 and 95% [7, 8]. The prevalence of WMSDs varies from region to region. According to the Bureau of Labour Statistics report, the annual prevalence of WMSDs in anybody region is between 40 and 85% among both Asian populations and Western populations [22]. In Nigeria, 84.4% of the nurses have had WMSDs once or more in their lifework [23]. Study conducted in Uganda indicated that 75% of nurses are vulnerable to musculoskeletal disorders because they work under highly resource resource-constrained ratio of 6:100,000 in Uganda is among the lowest worldwide, against the recommended WHO [24]. There is a direct association between understaffing, work overload, and the development of musculoskeletal disorders [20]. In Ethiopia as study done in Jima showed that the prevalence work-related musculoskeletal disorders at anybody site was 60% and the highest report 124 (67.8%) was seen in the lower back [25]. WMSDs are the most expensive form of work disability, attributing to about 40% of all costs toward the treatment of work-related injuries, and it has a high prevalence of 60% in developing countries, and 11% in developed countries [20]. The impact of W MSD results in nurses taking excess leave, quitting the profession, loss of working time, and loss of quality of life [9]. This means that WMSD leads to an increase in workforce and economic loss to health care institutions. Thus, the consequences of WMSD generate not only suffering and disability for workers and their families, but also result in excessive costs for society, considering losses in productivity and wages, benefits paid to workers, and medical expenses [20]. The challenges are also possibly the leading causes of poor patient outcomes, work demoralization, early retirement among nurses, and shy away from prospective nurses from the health sector. However, to our knowledge, there is little information on work-related musculoskeletal disorders among nurses in the study area. Accordingly, this study aimed to investigate whether there is statistically difference in work-related musculoskeletal disorders among nurses, considering a set of variables within the current study. The aim of the present study was to assess the magnitude of work-related musculoskeletal disorders and its factors associated among nurses working in public Hospitals of West Shoa Zone.

## Methods

### Study area and period

The study was conducted in West Shoa Zone, from March 2021 to June 2021. West Shoa is one of the 22 Zones of the Oromia Regional State in Ethiopia. Ambo city is the capital of West Shoa Zone located 111 km to west of Addis Ababa. Based on the information from West Shoa Zone Health Bureau, the population projection of 2014/15 of the Zone was 3 090 112 and Ambo town 184,925925 respectively. The Zone has a total area of 119,316 km2. In this Zone, there are eight Public Hospitals (Ambo University Teaching Hospital (AUTH), Ambo General, Guder, Gedo, Bako, Jaldu, Gindeberet, and Adaberga District Hospitals). Of the eight Public Hospitals, the first two are in Ambo town, the capital city of the Zone, whereas the other six are found in the six woredas of the west Shoa Zone, which are different distance from Ambo town. Except for AUTH which is a teaching Hospital, the seven are districting Hospitals. In addition to this the Zone has 520 health posts, 92 health centers and Ambo town administrative has 2 health centers. There are 498 enrolled nurses in West Shoa Zone Public Hospitals.

### Study design

An institution-based cross-sectional study design was performed.

### Source and study population

All nurses who were working in public Hospitals of West Shoa Zone were source population and all randomly selected nurses who are working in public hospitals of west Shoa Zone were study population. All nurses who had greater than12 months’ work experience were included in the study while nurses who are critically ill during data collection period, pregnancy mother and disabled were excluded from the study.

### Sample size determination

The sample size was calculated by using a single population proportion formula. To calculate the sample size prevalence rate of work-related musculoskeletal disorders was taken as 60% from the research done at Jima Zone of Southwest Ethiopia [26].

$${\rm{n = }}\frac{{{{\rm{Z}}^{\rm{2}}}{\rm{p }}\left( {{\rm{1}} - {\rm{p}}} \right)}}{{{{\rm{d}}^{\rm{2}}}}}$$

Where, *P* = prevalence of work related musculoskeletal disorders = 60%, Z = Level of confidence interval 95%=1.96, d = Degree of precession (the margin of sampling error to be used = 0.05.

$${\rm{n = }}\frac{{{{\left( {{\rm{1}}{\rm{.96}}} \right)}^{\rm{2}}} * {\rm{ 0}}{\rm{.60}}\left( {{\rm{1 - 0}}{\rm{.60}}} \right)}}{{{{\left( {{\rm{0}}{\rm{.05}}} \right)}^{\rm{2}}}}}{\rm{ = 369}}$$

By considering a 10%, non-response rates the final sample size was 406.

### Sampling technique

In the West Shoa Zone, there are eight Hospitals namely: - AURH, Ambo General, Guder, Gedo, Gindeberet, Jaldu, Bako, and Inchini Hospital with a total of 498 Nurses. Purposely all eight hospitals are included in this study and by simple random sampling 406 nurses were included in the study. The distribution of nurses was taken by population proportion size from the eight Hospitals: Ambo General Hospital has a total of 81nurses, Guder Hospital has a total of 52 nurses, Gedo Hospital has a total of 50 nurses, Jaldu Hospital has a total of 35 nurses. Gindeberet Hospital has a total of 50 nurses, AURH Hospital has a total of 150, Bako Hospital has a total of 30 nurses, and Inchini Hospital has a total of 50 nurses. To proportionate the number of study subjects for each hospital, the formula, n = n x nf/N was used.

### Variables

Dependent variable was work-related musculoskeletal disorders and independent variables were socio-demographic characters, psychosocial, work history factors, working environment, physical factors, and individual factors.

### Definitions of operational terms

Work-related musculoskeletal disorders (WMSDs) are defined as the discomfort reported by participant at least once in the last 12 months in one or more body region (wrist /hand, elbow, shoulder, neck, low back, knee, and ankle/feet).

Awkward postures: - means that the body’s natural alignment is out of its original position.

Physical factors: - are the aspects of the job or tasks that contribute to the development of MSDs when they impose a biomechanical (biological systems) stress on the worker [4].

Psychosocial factors: - are non-biomechanical risk factors related with work.

### Data collection tools and procedures

To collect data from study participants, a standard self-administered questionnaire was used. After a study of other relevant research, the data gathering tool was modified [6, 15, 19, 25, 27–33]. The questionnaires were prepared in English, and arranged according to the objective it addresses. It included four sections. Section (A): included the socio-demographic data and work history such as age, sex, height, weight, duration of work, position, salary, BM, and work settings. The WHO international classification of adults underweight, overweight, and obese according to BMI was adopted, as underweight (34). Section (B): to detect the prevalence of WMSDs among the studied nurses in the last 12 months, it was a symptom survey tool prepared from the Musculoskeletal Discomfort Form (Based on the Nordic Questionnaire) [12]. It shows a body chart of nine anatomic areas which are the neck, upper back, lower back shoulders, elbows, wrists/hands, hips/thighs, knees, and ankles/feet, and enquire about the existence of any troubles (pain and discomfort) during the last 12 months prior to the study. Section (C): includes data related to work-related factors that might be a factor in the development of WMSDs Section (D): includes individual factors.

Data were collected by ten BSc nurses’ data collectors and five master’s supervisors of health professionals were recruited. Three days’ training was given for the data collectors and supervisors on the study’s purpose, details of the questionnaire, and confidentiality of the respondents. After obtaining written consent the facilitators administered the questionnaire to the participants, collecting the questionnaire after the participants finished and submitting the filled questionnaire to the principal investigator timely.

### Data quality assurance

The quality of the data was assured through careful design and pretesting of the questionnaire using 5% of the total sample size at Tulu Bolo Hospital. Proper training of the interviewers and supervisors closes the data collectors’ supervision and proper handling of the data. The data was monitored frequently in the field and during the entry period to check completeness and consistency during interview and at the end of each day. Data entry was also done carefully by the principal investigator.

### Data processing and analysis

After data collection, each questionnaire was coded separately, and data was cleaned and input into the computer software EPI -info version 7.1 by the principal investigator. Prior to analysis, numerous variables were coded, particularly utilizing the computer software SPSS program version 25. For each variable, the frequencies, means, and standard deviation were calculated and displayed in the tables. To examine the relationship between the explanatory and outcome variables, contingency tables were used. In multivariable logistic regression analysis, variables with *p*-values less than 0.25 in bivariable logistic regression analysis were included. The *p*-value of 0.05 was deemed significant. By adjusting for the effect of, an odds ratio with a 95% confidence interval and logistic regression were used to describe the strength of relationship between explanatory and outcome variables.

### Ethical consideration

An ethical clearance was obtained from the Research Ethical Review Committee of Ambo University under the reference number AU/T512/2/17/2021. A letter of support was sent to public health institutions and other relevant organizations in the study area. Written informed consent was obtained from the respondents. Similarly, they were told of the study’s purpose, that it will contribute necessary information for policymakers and other concerned bodies to look after nurses in the study area at large. They were also told that all information obtained from them would be kept confidential and meant only for study. Privacy was also assured during the interview as some study questions are related to sensitive issues and an interviewee has been informed that he or she has the right either to be involved or not in the study.

## Results

### Socio-demographic characteristics of the participants

The response rate of this study was 397(97.8%). A total of 248(62.5%) males and 149(37.5%) females participated in this study. The minimum age of the respondents was 23 years, a mean age of 30.85 ± 6.1 years, and a maximum of 55 years old. This study revealed that 317(79.8%) of the participants were BSc holders. More than half of the participants were nurses with less than 5 years of work experience. In addition, 244 (61.5%) were married and 153 (38.5%) of them were single (Table 1).

**Table 1:** Socio-demographic characteristics of nurses working in West Shoa zone, Public Hospitals, Oromia Region, Ethiopia, 2021 (*N* = 397)

Variable	Categories	Number (%)
Gender	Male	248(62.5)
	Female	149(37.5)
Age	20–30 years	269(67.8)
	31–40 years	112(28.2)
	41–50 years	14(3.5)
	> 50years	2(0.5)
Marital status	Single	153(38.5)
	Married	244(61.5)
Level of education	Diploma	62(15.6)
	Bachelor	317(79.8)
Work experience	Masters	18(4.5)
	1-5years	283(71.3)
	6-10years	83(20.9)

### Prevalence of work-related musculoskeletal disorders

**Fig. 1:**
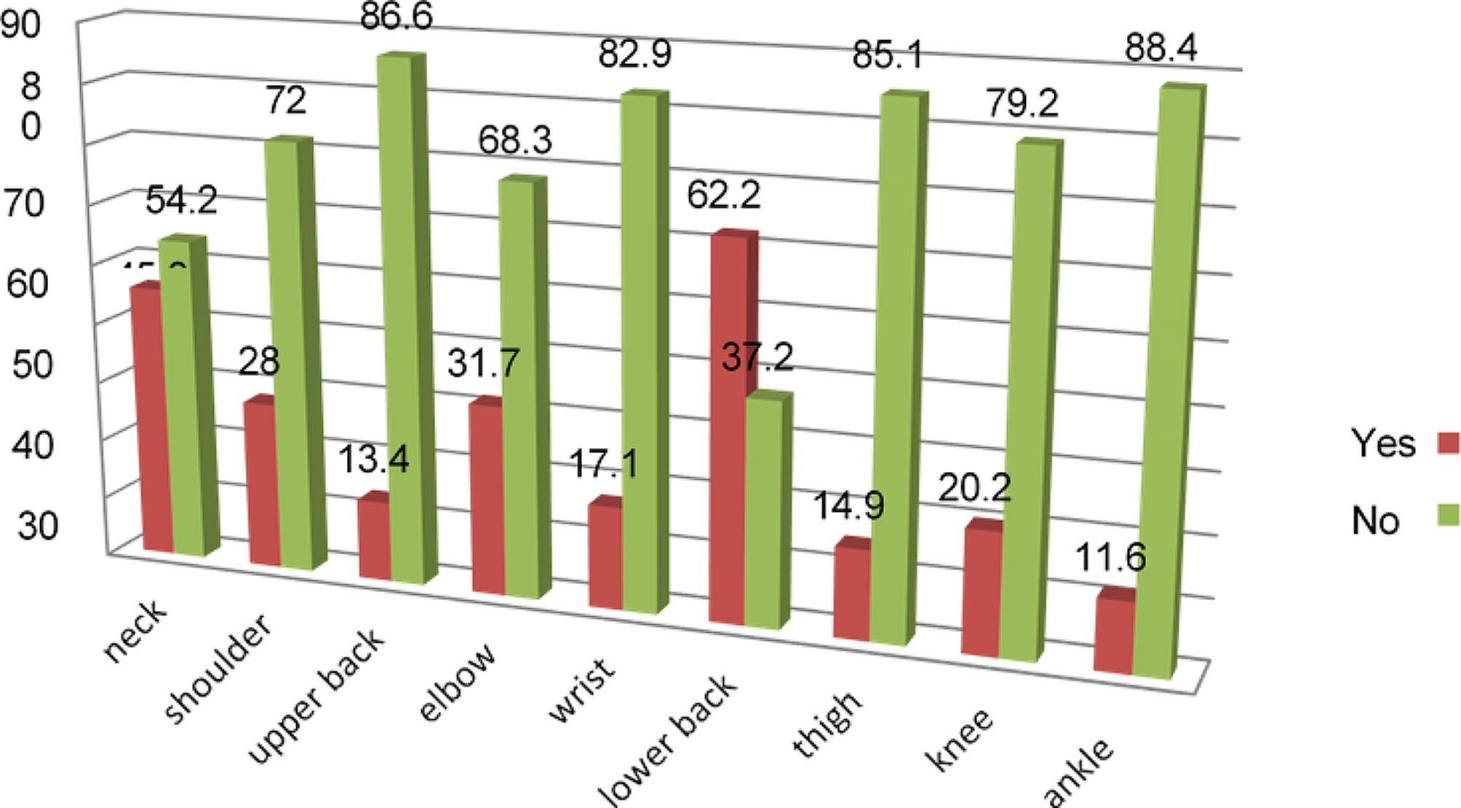
Prevalence of work-related musculoskeletal disorders in different body parts, 2021

The result of the study revealed that most nurses 73.8% [293] suffered from work-related musculoskeletal disorders in at least one region of the body in the last 12 months. Among them the 181 (45.6%) of males and 112 (28.2%) females. According to the current study the four most affected body regions that the respondents reported pain or discomfort in or around were the lower back 62.2% (*N* = 247), neck 45.8% (*N* = 182), elbow 31.7% (*N* = 126), and shoulder 28% (*N* = 111), respectively, while the lowest affected body part that the respondents reported was ankle/foot 11.6% ((*N* = 46) (Fig. 1).

### Organization and workplace factors

The majority of 225 (56.6%) participants reported that they have been working eight hours one standard shift on a typical day and 238 (58.7%) of them were working on rotational working shifts. Regarding their working unit 99 (25%) had been working in a medical ward, 81(20.4) were a working in surgical ward, 52 (13.1) were working Operation room, 67 (16.9) followed with those working in pediatric ward and the least was those who working in Psychiatry Room 11(2.8% with the number of patient attended by each respondents per a day, majority of the respondents, 286 (72%) attended 15–30 patients per/day, 61 (15.4%)o attended 31–45 patients per/day and the least was 17 (4.3%) who attended > 60 patients per/day respectively. Regarding periodical medical checkups 324 (81.6) of them had occasional medical check-ups. While on. While only 29 (7.3) had every six months medical checkup. Among the participants, only 22(5.54) have adequate on-the-job training on injury prevention (Table 2).

**Table 2:** Work characteristics of nurses who are working in West Shoa Zone Public Hospitals, Ethiopia, 2021(*n* = 397)

Variable	Categories	Number (%)
Working Unit/department	Medical ward	99 (25)
	Surgical ward	81(20.4)
	Pediatrics Ward	52(13.1)
	Neonatology Ward	17(4.3)
	ICU	33(8.3)
	Operation Room	67(16.9)
	Psychiatry Room	11(2.8)
	OPD	37(9.2)
Number of patient attended by the respondents per a day	15–30	286(72)
	31–45	61 (15)
	46–60	33(8.3)
	> 60	17(4.3)
Working hours/day	8 h	225(56.7)
	8–12 h	125(31.5)
	> 12 h	47(11.8)
work shift	Constant	164(41.3)
	Rotational	233(58.7)
Predicable medical check up	Every six months	29(7.3)
	Every years	34(8.6)
	Every two years	10(2.5)
	Occasionally	324(81.6)
Status of on job training on injury prevention	Have no training on injury prevention	277(69.8)
	Have no adequate training on injury prevention	98(24.7)
	Have adequate training	22(5.5)

### Psychosocial and behavioral factors

Based on the working environment, the study participants were asked about their relationship with other colleagues 102 (25.7%) of them reported that they had a poor work relationship with their colleagues and supervisors. The majority of 180(45.3%) of the respondents said that they have poor work rest/cycles. Regarding high-loaded work, 240 (60.5%) of them had a high workload and 120 (30.2%) had a lack of control were reported, respectively. The result indicated that 47.6% (*N* = 189) of respondents participated in leisure activities. Regarding alcohol use, 15.7% (*N* = 62) of the respondents drank alcohol. The result also indicated that 23.9% (*N* = 95) of respondents practiced housekeeping activities. More than half of the participants, 79.8% (*N* = 317) had a BMI in the normal range (Table 3).

**Table 3:** Showed that the working environment, psychosocial/behavioral factors among the nurses working in the public hospitals of West Shoa Zone in 2021 (*N* = 397)

Variables		Number (%)
Psychological factors	Poor Working relationship with supervisors and colleagues	102(25.7)
	financially demoralized	88(22.2)
	poor work/rest	180(45.3)
	poor community support	27(6.8)
	High work load	240(60.5)
	Tight deadline	37(9.3)
	Lack of control	120(30.2)
Personal / individual factor	Body mass index <18 kg/m2 18–24.9 kg/m^2^ 25–29.9 kg/m^2^ =>30 kg/m2	35(8.8)
		317(79.8)
		43(10.8)
		2(0.5)
	Educational background Diploma BSc Masters	62(15.4)
		317(79.8)
		18(4.5)
Behavioral factors	Alcohol consumption	62(15.6)
	Smoking	51(12.8)
	Leisure activities	189(47.6)
	House keeping	95(23.9)

#### Physical factors related with work-related musculoskeletal disorders

Based on the neck position they used, 288 (72.5%) of the participants reported that they had bent their neck forward when working, 66(16.6) of them used backward and 43 (10.8%) had used lateral/twisting positions during their work activities. Regarding their working schedule the majority of 285(71.8%) respondents reported that they were working mixed time. As the respondents asked whether their work required repetitive motion 195 (49.1%) of them reported that their work had required repetitive motion. Additionally, the respondents were asked about lifting heavy loads during their working time 227 (57.2) of them lifted heavy loads during their work time. Also, the participants were asked whether they used awkwarp positions based on this 253(63.5) were used but the rest were not (Table 4).

**Table 4:** Physical factors related with work related musculoskeletal disorders among participants working in west Shoa in 2021(*N* = 397)

S/*n*.	Variable			Numbers	Presents	
	Working schedule	Day		99(24.9)		
		Night		13(3.3)		
		Mixed		285(71.8)		
	Neck position	Bending forward		288(72.5)		
		Back ward		66(16.6)		
		Lateral/twisting		43(10.8)		
	Repetitive position			195(49.1)		
	Lifting heave loads			227(57.2)		
	Use Awkward position	Yes	253(63.5)			
		No	145(36.5)			

### Factors associated with work-related musculoskeletal disorders

Binary and multivariable Logistic regressions were performed to assess the association of each independent variable with WRMSDS. Working in the medical ward, 5.91 times more likely (COR = 5.91, 95%CI: 2.53–13.79), surgical ward, 9.63 times more likely (COR = 9.63, 95%CI: 3.64–15.51), and operation room theater 6.02 times more likely (COR = 6.02, 95%CI: 2.37–15.27) had higher risk to work-related musculoskeletal discomfort as compared to those who were worked in outpatient department. Nurses those experienced bending or twisting back as a problem during work 2.33 times more likely to have higher risk WMSDs as compared to those who didn’t perceive it with (COR 2.33, 95% CI: 1.47–3.71). Regarding which neck position they used, the participants who had used bending forward position were 16.13 time more likely at risk to develop WMSDs with (COR = 16.13, 95%, CI: 7.78–14.38) than as compared with those who had used bending lateral position. Participants who have less working experience are 9.27 times more likely at risk for developing WRMSDs with (COR = 9.27, 95%CI: 3.10-17.71) when compared with those who have more working experience. Participants who had no job training for injury prevention were 5.5 times more likely to develop WMSDs with AOR = 5.52, 95%CI: 2.26–13.51) than those who had adequate on-job training for injury prevention (Table 5).

**Table 5:** A multivariable logistic regression analyses of the various risk factors for reported WMSD among Nurses Working in West Shoa Zone Public Hospitals, South West Ethiopia, in, 2021

Variable	Category	WRMSDs		OD ratio	Adjusted OD ratio	*P* value	
		yes	No				
Working unit	Medical	84(84.85%)	15(15.15%)	5.91(2.53–13.79)	2.43(0.81–7.24)	0.112	
	Surgical	73(90.12%)	8(9.88%)	9.63(3.64–15.51)	10.48(3.02–16.37)	0.000	
	OR	57(85.07%)	10(14.93%)	6.02(2.37–15.27)	3.02(0.88–10.38)	0.080	
	Outpatient department	18(48.65%)	19(51.35%)	1	1	1	
Marrital status	Married	217(88.93%)	27(11.07%)	0.12(0.07–0.21)	0.24(0.12–0.46)	0.001	
	Single	76(49.67%)	77(50.33%)	1	1		
Working in the same positions for long period	Has problem	171(72.15%)	66(27.85%)	0.81(0.51–1.28)	0.92(0.43–1.20)	0.363	
	No problem	122(76.25%)	38(23.75%)	-	-	1	
Performing the same task over and over(less than 2 s)	Has problem	145(74.36%)	50(25.64%)	1.06(0.70–1.66)	1.27(0.59–2.42)	0.035	
	No problem	148(73.27%)	54(26.73%)	1	-	1	
Work experience	1-5years	198(70%)	85(30%)	1.68(0.79–3.59)	1.24(0.40–3.87)	0.721	
	6-10years	77(92.8%)	6(7.2%)	9.27(3.10-17.71)	9.31(2.11–18.06)	0.003	
	≥ 11years	18(58.1%)	13(41.9%)	1	1	1	
Does your job involves bending or twisting in an awkward way?	Has problem	165(81.68%)	37(18.32%)	2.33(1.47–3.71)	0.44(19 − 0.90)	0.016	
	No problem	128(65.60%)	67(34.4%)	1	1		
Which neck potion do you use	Bending forward	258(90.2%)	28(9.8%)	16.13(7.78–14.38)	12.45(4.95–12.32)	0.000	
	Bending back ward	19(28%)	48(72%)	0.69(0.31–1.56)	0.59(0.22–1.64)	0.327	
	Bending lateral	16(36%)	28(64%)	1	1	1	
Status of on job training on injury prevention	No training	236(89.1%)	29(10.9%)	13(6.60-25.67)	5.52(2.26–3.51)	0.002	
	inadequate training	37(46.3%)	43(53.7%)	1.48(5.27–6.97)	2.52(0.97–6.61)	0.059	
	Have adequate training	20(38.5%)	32(61.5%)	1	1	1	

## Discussion

The current study revealed that work-related musculoskeletal disorders in any region was 73.8%, which showed that many nurses in the study area suffered from work-related musculoskeletal disorders last year (12 months). This might cause less patient care due to nurses feeling discomfort and even being absent from their duty, taking leave, and while on their working day, they did not feel free to perform their daily tasks. The result of this study showed that it is slightly less than the study done in Nigeria, South Africa, Kenya, and Uganda (84%, 80.8%, 79% and 75%) respectively [13, 23, 24, 35]. The discrepancy might be due to the sample size used in each study area not being the same, the study period, and the variation in nursing tasks and procedures in different countries.

This study’s finding was lower than the prevalence of the previous studies such as a study done in 2015 in Zambia found a prevalence of 68.9% and Jima 60% [25]. These differences in the prevalence may be due to work overload, and inadequate job training for injury prevention that predisposing nurses in our study who face too much occupational risk factors related to their stressful heavy work that make them at higher risk to develop WRMSDs than nurses working in other study area.

Based on the body site the height prevalence in this study was lower back pain (62.2%), neck pain (45.8%), elbow pain (31.7%), shoulder (28%), and knee pain (20.2%) respectively. This is higher than the study done in South Africa [36], and Ibadan [23] but, less than the study done in Kenya, Zimbabwe, and Jima (25, 37, 38). In the entire study area, low back pain is the most prevalent problem among the study participants. This could be due to the lack of use of appropriate way of body mechanics which involves the coordinated effort of muscles, and bones, to maintain balance, posture, and alignment, like during the moving, transferring, and positioning patient, if the nurse did not work as close to an object to be lifted as possible in order to decrease their effort. The annual prevalence of neck pain in South Africa (21%), Uganda (25.93%), and Jima (24%) were less than the current study. this discrepancy may be due to the current study was done on more distinct levels of the hospital, using the more manual ways of lifting, and moving equipment and patients rather than using mechanical methods. According to the current study, work-related musculoskeletal injury or discomfort caused negative effects on the daily activities of the respondents. About 92(23.3%) of respondents were absent from their job at least eight days, because of they felt discomfort. Regarding the WRMSDs risk factors, the present research reported that the most prevalent risk factors were, working in the same positions for extended periods 171(43.1%), performing the same task repeatedly (36.5%), followed by neck position (bending lateral position) 16(4%). This is consistent with the study done in some areas like Nigeria and the study done in Jimma, Ethiopia showed that occupational factors such as working unit, working in the same positions for prolonged periods, working in malposition, and bending or twisting back during work were factors significantly associated with WMSDs among nurses. Among the risk factors that were significantly associated with work-related musculoskeletal disorders marital status (married) 217(54.7) and having no job training for injury prevention 236 (59.9%) were the other factors significantly associated with WMSDs among nurses. This might indicate that there is a problem/gap in the study area on the on-the-job training for nurses about injury prevention or that the use of principles of body mechanics is extremely poor.

## Conclusions

The prevalence of work-related musculoskeletal disorders among nurses was high in the study area. In this study factors such as those having no on-the-job training about injury prevention, marital status, working care unit, working in malposition, working in the same positions for prolonged periods of time, and bending or twisting back during work were identified as associated factors. Since WMSDs are preventable, educating the staff about the use of proper body mechanics and modifying the work environment is essential for musculoskeletal disorders. Working environments for patient handling require floors that are even, non-slip, and stable. The workload should be organized in such a way as to minimize manual patient handling operations and provide furniture and equipment that effectively reduce handling operations, to distribute handling tasks evenly throughout the shift, and allow the personnel adequate rest and recovery periods.

## Data Availability

The datasets used and/or analysed during the current study available from the corresponding author on reasonable request.
